# A Missing Voice? Peer Support Workers’ Perceptions of Psychedelic‐Assisted Therapy in Australia: A Cross‐Sectional Survey

**DOI:** 10.1002/brb3.71521

**Published:** 2026-05-31

**Authors:** Aloysius Amos Lau, Shalini Arunogiri, Boen Raner‐Galutera, Siegfried Lichtwark, Sarah J. Catchlove

**Affiliations:** ^1^ Eastern Health Clinical School Monash University Melbourne Australia; ^2^ Turning Point, Eastern Health Melbourne Australia

**Keywords:** attitudes, mental health, peer support, psychedelic‐assisted therapy, substance use disorders, survey

## Abstract

**Introduction:**

Peer Support Workers (PSWs) in mental health and substance use treatment draw on their lived experience (LE) to support clients through mutual understanding and trust. Psychedelic‐assisted therapy (PAT) is emerging as a mental health and substance use treatment modality. As interest grows, understanding PSWs’ perspectives on PAT is critical to inform safe and effective implementation, and the potential role PSWs can play in models of care. This study examined factors shaping PSWs’ perspectives toward PAT and perceived barriers to its adoption and implementation.

**Methods:**

A mixed methods design was employed. A cross‐sectional survey informed by the Theoretical Domains Framework (TDF) captured PSWs’ perceptions of PAT and potential barriers to its use. This was followed by semi‐structured interviews with five survey participants. Interview transcripts were analyzed to provide a qualitative description and identify topics and patterns to enrich and contextualize the survey findings.

**Results:**

Survey responses indicated strong PSW support for PAT, with most participants expressing willingness to recommend it to clients. Qualitative findings expanded on this interest, highlighting concerns around client safety, psychoeducation, stigma, and accessibility. The qualitative description of interview results identified key influences on PSWs’ attitudes toward PAT, complementing survey results and aligning with existing literature.

## Graphical abstract

Despite their capacity to bridge providers and clients, peer support workers remain largely absent from Australia's emerging psychedelic‐assisted therapy (PAT) landscape. This study identifies a broadly supportive and optimistic PSW workforce, while highlighting the need for tailored education pathways and structured integration models to enable effective involvement in developing PAT frameworks.

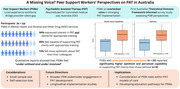

## 1 Introduction

Mental health (MH) and substance use disorders (SUDs) contribute significantly to the global health burden, affecting over one billion people, conducive to an estimated economic cost exceeding 4.7 trillion USD in 2019 (Arias et al. 2022; Rehm and Shield 2019). Despite advancements in intervention strategies, MH and SUDs still account for a large share of the global burden of disease (GBD 2019 Mental Disorders Collaborators 2022). Relapse is particularly common in SUDs, with an estimated 40%–60% of individuals returning to substance use within a year of completing treatment (Beaulieu et al. 2021).

Peer support involves individuals with lived experience of MH or substance use challenges providing social and emotional support to foster personal recovery (Fortuna et al. 2022). In Australia, peer support is recognized as a key component of the national mental health strategy (National Mental Health Commission 2017). This policy reflects a growing recognition that relational and experiential forms of support can complement traditional clinical care. Negative attitudes among healthcare providers often reinforce stigma and hinder treatment outcomes (Knaak et al. 2017; van Boekel et al. 2013). Such stigma can widen the divide between providers and service users, reducing engagement and trust in formal services. Peer support workers (PSWs), through their lived experience, can bridge this provider‐client gap by building trust, modelling recovery and supporting transitions to community‐based care (Lennox et al. 2021). While evidence highlights their role in reducing stigma and enhancing engagement, clear frameworks are needed to guide implementation (Orock and Nicette 2022; Tracy and Wallace 2016), especially in the context of emerging treatment modalities.

Psychedelic‐assisted therapies (PAT) are one such emerging modality, as promising interventions for treatment‐resistant MH and SUDs, with global research growth, hundreds of trials internationally and over 30 registered locally on the Australia New Zealand Clinical Trials Registry (ANZCTR) (July 2025). In July 2023, the Therapeutic Goods Administration (TGA) reclassified psilocybin and MDMA for controlled medical use, marking a significant regulatory milestone (Therapeutic Goods Administration (TGA) 2023). While early evidence shows strong promise for PTSD and depression (Cavarra et al. 2022), studies are expanding to investigate their role in SUDs. Retrospective studies suggest psychedelics may reduce substance dependence (Zafar et al. 2023), and numerous randomized controlled trials are now underway to formally investigate their therapeutic potential in SUDs (Kurtz et al. 2022).

In Australia, the approved delivery model for PAT in both clinical and research settings involves a co‐therapist dyad of licensed health professionals (RANZCP, 2023). This is a notable departure from the community and group‐based settings in which psychedelics have traditionally been used (Trope et al. 2019). This intensive and costly model presents significant challenges to scalability and accessibility. A course of PAT under this protocol is expected to cost AU$25,000–AU$35,000 (Chrysanthos 2023), making it largely inaccessible outside of clinical trials.

As efforts to scale PAT continue, alternative delivery models such as group‐based protocols are being explored, partly to address issues of accessibility and cost. In parallel, community‐led psychedelic peer support initiatives—pioneered in the 1960s and exemplified by organizations like The Zendo Project and the Fireside Project—have long provided harm reduction and integration support for individuals who use psychedelics in non‐clinical settings (Skiles et al. 2023). In the US state of Oregon, regulated psilocybin services have created pathways for non‐licensed peer specialists, whose roles involve case management or co‐facilitating groups in a type of community‐based integration (Skiles et al. 2023). While these initiatives operate outside formal medical frameworks, they illustrate how lived‐experience‐informed support may enhance perceived safety, cultural responsiveness, and relational trust, particularly in mitigating risks associated with naturalistic psychedelic use. These precedents provide a conceptual basis for considering how PSWs may be integrated within Australia's emerging PAT framework to enhance relational support across clinical and community contexts. However, little is known about how PSWs themselves perceive these roles within Australia's evolving PAT landscape.

Healthcare providers’ attitudes toward PAT vary considerably across settings and disciplines, reflecting both enthusiasm and concern. Despite the cost of PAT, psychologists in the U.S. and New Zealand expressed active interest in the therapy (Reynolds et al., 2022), emphasizing the need for increased research, noting stigma as a hindrance to trial recruitment and expansion of knowledge in the area. Providers who were more familiar with the legalities and risks of using psychedelic substances held more favorable attitudes toward PAT, describing it as “having a major place [in treatment]” (Kucsera et al. 2023; Luoma et al. 2023). In contrast, psychologists who were less informed often dismissed PAT as “crazy” and “without evidence” (Luoma et al. 2023). British psychologists echoed these concerns, expressing uncertainty about adverse effects, despite evidence of a strong safety profile. Client safety and wellbeing are common concerns in the literature. Palliative care workers reflected this divide: while there was concern regarding possible risks, others supported PAT as a modality to alleviate patient suffering (Niles et al. 2021).

While perceptions of PAT have been explored among numerous stakeholders, including psychiatrists, psychologists, consumers, and policy makers (Barnett 2018; Kunstler et al. 2023; Nadeem et al. 2024; Negrine et al. 2025), little is known about the views of PSWs. This gap is notable, given their expanding role within Australia's mental health (MH) and alcohol and other drugs (AOD) treatment workforce, and their unique capacity to foster trust, reduce stigma, and enhance treatment engagement. As frontline providers with lived experience, PSWs are positioned to play an important role in shaping equitable access to emerging treatments such as PAT. Understanding their views is therefore essential to guide both service development and policy. The present study aims to address this gap by investigating PSWs’ attitudes toward PAT using an implementation theory‐informed approach, examining factors that shape their recommendations to clients and identifying perceived barriers to implementation within the Australian context.

## 2 Methods

### 2.1 Study Design

This study employed a mixed‐methods design, consisting of a quantitative cross‐sectional survey and a subsequent qualitative extension with semi‐structured interviews (see Supplementary Material 1 for results). Ethics approval was obtained from the Eastern Health Human Research Ethics Committee (Reference No: E24‐001‐105296). The qualitative component was added retrospectively to gather more detailed insights. Data collection occurred between March and September 2024.

### 2.2 Participants

Individuals who self‐identified as being currently employed as a MH or AOD peer support worker in Australia were eligible. Participants were invited to complete the anonymous online survey and were given the option to provide their e‐mails to the research team if they desired to take part in the qualitative section of the study.

Recruitment was conducted via snowball sampling. A survey link was distributed through professional mailing lists from participating MH, AOD and lived experience services, organizational networks, and social media (LinkedIn, X, etc.). From an initial pool of *N* = 59, *n* = 33 responses were retained for the final quantitative analysis. 27 responses were excluded for either not meeting eligibility criteria (*n* = 20) or providing incomplete data (*n* = 7). Five eligible participants consented to and completed the supplementary qualitative interview (included in Supplementary Material 1). Participation for both components of this study was voluntary and uncompensated.

### 2.3 Measures

The questionnaire included four main components: (i) participant demographics (age, sex, level of education, primary role, years of experience in the role, lived experience with MH and/or SUDs, geographical location, prior psychedelic experience); (ii) attitudes to PAT; (iii) personal psychedelic experience (pertaining to set and setting and perceived positive outcomes if applicable); and (iv) desired educational topics.

The Theoretical Domains Framework (TDF) brings together constructs from 33 behavior change theories into 14 domains, to examine the cognitive, emotional, social, and environmental factors that shape health professionals’ implementation of evidence‐based practices (Atkins et al. 2017). This project used a TDF‐informed questionnaire adapted from a study assessing attitudes of MH and AOD clinicians toward PAT (Bryant et al. 2025). Of the 14 domains outlined by the TDF, six were selected based on their theoretical relevance to understanding attitudinal, motivational, and contextual factors influencing PSWs’ support for PAT: (i) knowledge, (ii) social/professional role, (iii) beliefs and optimism, (iv) beliefs about Consequences, (v) motivations and goals, and (vi) barriers and facilitators. The attitudinal items were rated on a five‐point Likert scale (1 = Strongly Disagree to 5 = Strongly Agree). The TDF‐informed items underwent revisions by the study team and were reviewed by an external subject matter expert. The final 74‐item questionnaire was administered in the online survey, and the completion time was approximately 20 min.

### 2.4 Data Analysis

Quantitative data was analyzed using R (v 4.4.1). The analysis focused on descriptive statistics and an exploration of inter‐domain relationships.

In the absence of a validated instrument for this context, TDF‐informed items were adapted to ensure relevance and clarity while retaining theoretical alignment. This allowed the development of attitudinal domains that were conceptually coherent and psychometrically sound. Items that did not demonstrate clear conceptual alignment with an attitudinal domain or that reduced a scale's internal consistency were excluded. Domain scores were calculated by averaging the item scores within each domain.

**FIGURE 1:**
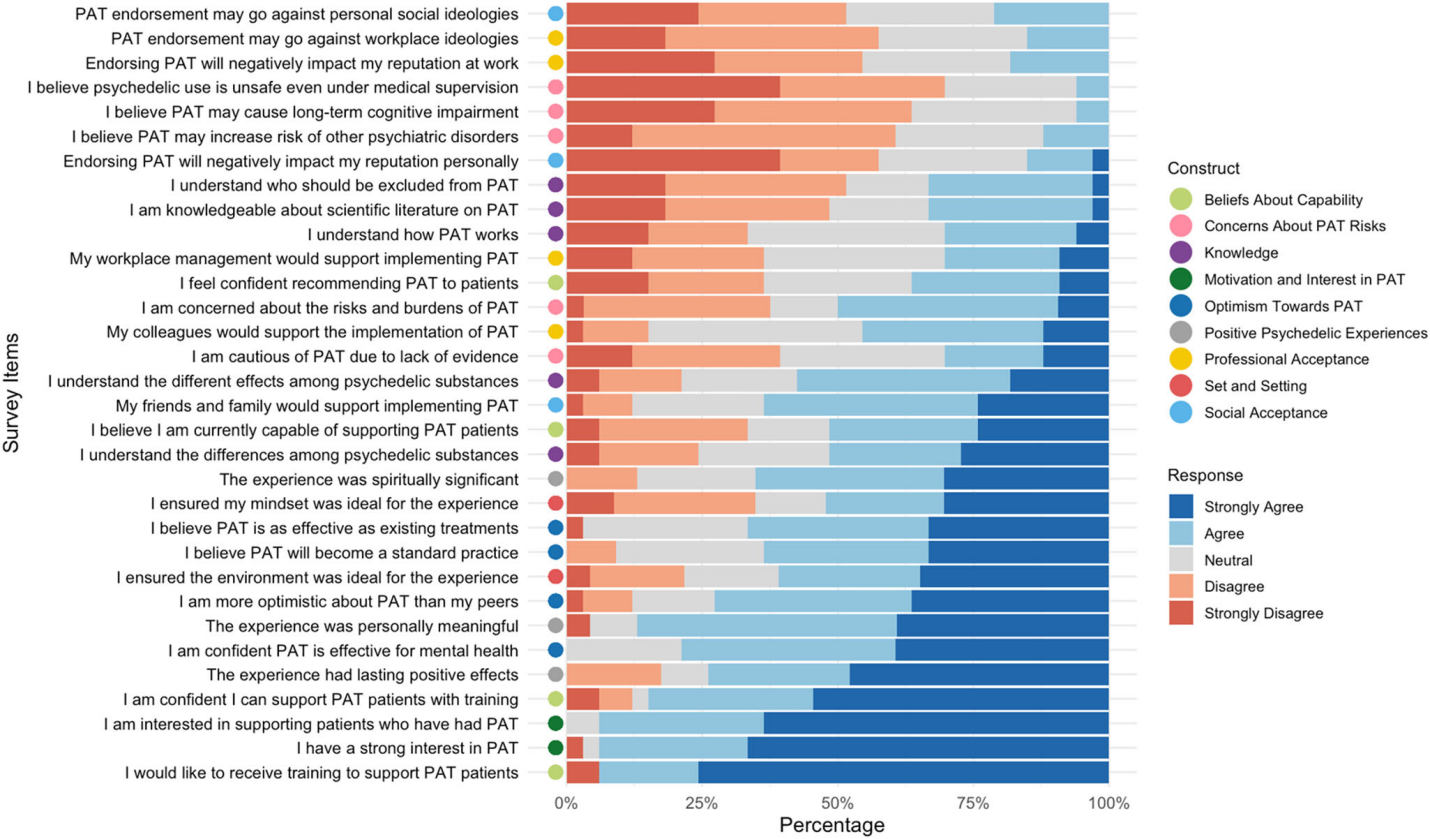
Percentage distribution of participant responses for each survey item. Items are ordered from lowest to highest overall agreement. Survey item wording has been abbreviated in this figure for brevity.

This process resulted in nine distinct domains, all of which achieved acceptable internal consistency (Cronbach's *α* > 0.70). Seven domains were derived from the TDF‐informed items: (1) Professional Acceptance of PAT (*α* = 0.83), (2) Social Acceptance of PAT (*α* = 0.77), (3) Beliefs About Capability (*α* = 0.74), (4) Optimism Toward PAT (*α* = 0.72), (5) Concerns About PAT Risks (*α* = 0.74), (6) Knowledge (*α* = 0.90), and (7) Motivation and Interest in PAT (*α* = 0.81). Two additional domains assessed perceptions of “Set and Setting” (Cronbach's *α* = 0.90) and “Positive Outcomes” (Cronbach's *α* = 0.82) relating to one's most impactful psychedelic experience. A descriptive summary of responses for the items comprising each construct is presented in Figure 1 (see Table S1 for full survey items), which is discussed further in the Results section. Due to the modest sample size, factor analysis for the domains was not performed.

Normality was assessed using the Shapiro–Wilk test to guide the choice of inferential test for inter‐domain analyses. Given the ordinal nature of Likert‐based scores and evidence of non‐normality in several domains, group comparisons were conducted using Wilcoxon rank‐sum tests. Effect sizes were expressed as Cliff's delta, which is robust for ordinal data and non‐parametric group comparisons. Inter‐domain correlation analyses were conducted using Spearman's rank‐order correlation. To adjust for inflated Type I error from multiple testing, the Benjamini–Hochberg procedure was applied to control the false‐discovery rate while preserving statistical power (Benjamini and Hochberg 1995).

## 3 Results

### 3.1 Demographics

Demographic characteristics of the survey participants are presented in Table 1. The mean age was 40 years (SD 9.6, range 22–61).

**TABLE 1:** Demographic characteristics of survey participants.

Demographic variables	Mean (± SD, range)	*n*	Percentage
**Gender**			
Male		9	27.3%
Female		23	69.7%
Not specified		1	3.0%
**Indigenous status**			
Yes		1	3.0%
No		32	97.0%
**Education level**			
Secondary		2	6.1%
Diploma		13	39.4%
Undergraduate degree		11	33.3%
Master's degree		5	15.2%
Other		2	6.1%
**Duration of peer support work experience**			
<6 months		4	12.1%
6–12 months		4	12.1%
1–3 years		10	30.3%
> 3 years		15	45.5%
**Primary area of peer support work**			
Client engagement		22	66.7%
Education		2	6.1%
Research		2	6.1%
Management		1	3.0%
Public health advocacy		2	6.1%
Other		4	12.1%
**Lived experience (yes)**			
Mental health		31	93.9%
Substance use disorders		26	78.8%
**Geographical location in Australia**			
Metropolitan		29	87.9%
Regional		4	12.1%

### 3.2 Descriptive Statistics

Responses to survey items of attitudinal domains are shown in Figure 1. Participants expressed strong enthusiasm for PAT: A majority (94%) reported interest in PAT, willingness to support individuals undergoing treatment, and a desire for appropriate training. Most (85%) agreed that with appropriate training and guidelines they would feel capable of providing support, and that greater knowledge would increase their willingness to recommend PAT to clients. Many endorsed the need for further research (90.9%), expressed confidence in its effectiveness for mental health disorders (78.8%), and considered it as effective as existing treatments (63.6%). Over two‐thirds (72.7%) reported greater optimism about PAT than their peers.

Participants strongly endorsed the need for further education regarding PAT. High levels of agreement were observed across all listed topics, including *management of challenging experiences (i.e., “bad trips”)* (93.3%), *pharmacology of psychedelics* (90%), *side effects of psychedelics* (86.7%), *contraindications for PAT* (86.7%), and *potential benefits of PAT* (86.7%) (see Table S1).

This enthusiasm contrasted with self‐reported knowledge gaps. About half of participants disagreed with understanding PAT exclusion criteria (51.5%) and PAT‐related scientific literature (48.5%), while a‐third reported limited understanding of how PAT works (33.4%).

Concerns about reputational harm were low. A majority disagreed that endorsement would negatively affect their professional (54.6%) or personal (57.6%) reputation while around one‐quarter remained neutral. A plurality believed colleagues would be supportive of PAT implementation (45.4%), though views of workplace management support were mixed.

### 3.3 Prior Psychedelic Experiences

**FIGURE 2:**
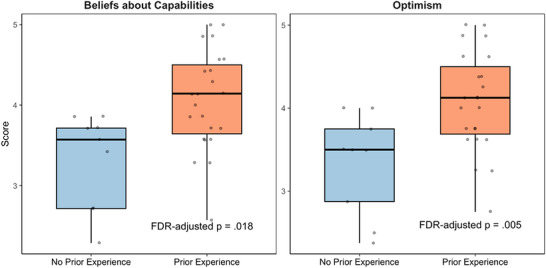
Comparison of “Beliefs about Capabilities” and “Optimism” domain scores between PSWs with and without prior psychedelic experience. FDR = false‐discovery rate.

One participant did not complete this section of the survey. Of the remaining 32 participants, *n* = 23 reported previous personal psychedelic use, while *n* = 9 did not (Figure 2). In addition, 21 participants (65.6%) reported having supported clients who had used psychedelics, and 28 (87.5%) indicated that close contacts (e.g., friends or family members) had personal experience with psychedelic substances.

Among respondents with personal psychedelic experience (*n* = 23), a majority reported their experience was personally meaningful (86.9%), had long‐lasting positive effects (73.9%), and was spiritually significant (65.2%). However, a secondary correlational analysis found no statistically significant associations between these self‐rated positive experiences and any of the primary attitudinal domains.

A statistically significant difference was found in the “Beliefs of Capability” domain (*W* = 41.5, adjusted *p* = 0.018). Participants with prior psychedelic use reported significantly higher confidence in their ability to support patients undergoing PAT (Mdn = 4.14) compared to those without lived experience (Mdn = 3.57). This difference represented a medium‐to‐large effect size (Cliff's Delta = 0.60, 95% CI [0.20, 0.83]). A statistically significant difference was found in “Optimism” wherein participants with prior psychedelic experience reported greater optimism toward PAT (Mdn = 4.12) compared to those without (Mdn = 3.50) (*W* = 37.5, adjusted *p* = 0.005). This difference represented a large effect size (Cliff's Delta = 0.64, 95% CI [0.25, 0.84]). There were no other statistically significant differences based on previous psychedelic experiences observed with the remaining domains.

### 3.4 Bivariate Associations

Correlational analyses (Table 2) between attitudinal domains revealed associations among perceived “*Knowledge*,” “*Optimism*,” and “*Beliefs about Capabilities*” in supporting PAT patients. “*Optimism*” and “*Beliefs about Capabilities*” showed the strongest relationship (*ρ* = 0.98, *p* < 0.001), while “*Knowledge*” showed significant positive correlation with both variables as well as with “*Professional Acceptance*.” These domains were negatively correlated with “*Beliefs about Risks*.”

**TABLE 2:** Spearman's correlation matrix between attitudinal domains.

Domains		1	2	3	4	5	6	7
**1**	**Knowledge**	1	0.45*	0.25	0.41*	−0.40*	0.43*	0.23
**2**	**Professional Acceptance**		1	0.62***	0.59**	−0.53**	−0.55**	−0.05
**3**	**Social Acceptance**			1	0.36	−0.40	0.36	−0.14
**4**	**Beliefs about Capabilities**				1	−0.63***	0.98***	0.18
**5**	**Beliefs about Risks**					1	−0.60**	−0.12
**6**	**Optimism**						1	0.21
**7**	**Motivation**							1

*p < 0.05.

**p < 0.01.

***p < 0.001.

## 4 Discussion

The present study investigated PSW's attitudes toward PAT using a theory‐driven approach and identified perceived barriers to implementation. This study provides the first known exploration of PSW perspectives on PAT in an Australian context. Our findings reveal a high degree of acceptance among PSWs, with key results demonstrating that prior lived experience with psychedelics and perceived knowledge was significantly associated with both higher perceived capability in supporting PAT clients and greater optimism toward PAT. Participants in this survey were not asked to specify whether their reported psychedelic experiences occurred in naturalistic or regulated clinical contexts. However, given the limited accessibility of formal PAT within Australia at present and the fact the survey was conducted less than 2 years after implementation of regulatory changes, it is likely that most reported experiences reflect non‐clinical use.

This pattern aligns with broader research into stakeholder attitudes. Internationally, self‐rated knowledge and personal experience are key predictors of public perception regarding psychedelic legalization, benefits, and risks (Žuljević et al. 2022). In Australia, a recent survey similarly found that in both MH service users and the general public, psychedelic experience and greater knowledge were more likely to hold positive views toward PAT (Nadeem et al. 2024). Results of the qualitative interviews reflect these trends, with participants noting that even challenging experiences gave them a greater understanding of the need for alternative treatments like PAT. A study assessing perceptions among Australian MH clinicians found that lived experience was linked to greater self‐efficacy, motivation, and more positive beliefs about the safety and benefits of PAT (Bryant et al. 2025). Collectively, these findings highlight knowledge and personal experience as key drivers of professional acceptance, underscoring the importance of education and inclusion of lived experience within emerging PAT frameworks.

The findings of this study should be interpreted in the context of several methodological limitations. First, the modest quantitative sample size (*N* = 33) reduces statistical power and limits the generalizability to the broader Australian PSW workforce. In specialized mental health services, there were approximately three full‐time peer support workers per 100,000 population in 2022–2023 (Australian Institute of Health and Welfare 2025). However, the size of the broader community‐based workforce remains difficult to estimate (Private Mental Health Consumer Carer Network (Australia) 2019). Recruitment via online snowball sampling without compensation likely introduced a self‐selection bias toward participants with pre‐existing interest in psychedelic therapies, reflected in 72.8% reporting greater optimism about PAT compared to their peers. The sample was skewed toward middle‐aged, female participants with high educational attainment and under‐represented Indigenous voices. Finally, while attitudinal constructs were theory‐driven and internally reliable, they were not validated instruments.

Strong interest in accessible education pathways for non‐clinician roles was evident in our findings. A large majority (94%) of participants expressed willingness to undertake PAT‐specific training and 85% agreed that more knowledge would increase their readiness to recommend it. Notably, the educational domains endorsed were practical and safety‐oriented—including management of challenging experiences, pharmacology, side effects, and contraindications—suggesting that PSWs are seeking concrete competencies rather than abstract endorsement of PAT. While previous studies have established therapist competencies as essential for effective PAT delivery (Phelps 2017), current training programs remain geared toward licensed clinical health professionals, excluding non‐clinician roles. Integrating PAT‐specific content into PSW educational programs could enhance the preparedness and impact of this workforce in supporting PAT interventions.

The findings provide future directions for implementing PSWs in PAT protocols. Qualitative data highlighted concerns regarding stigma, role ambiguity, and the high cost of PAT (see Supplementary Material 1). Leveraging PSWs may offer a solution to address gaps in current PAT models, particularly in providing post‐PAT integration support. Discontinuity of care post‐PAT in clinical trials is a major challenge, as therapeutic alliances may be disrupted (Jacobs et al. 2024). Continuity is critical, with therapeutic alliance predicting long‐term reductions in depression (Levin et al. 2024; Murphy et al. 2021). The therapeutic bond consolidated after the psychedelic experience is vital for sustaining long‐term therapeutic gains. Evidence from the broader peer support literature suggests that peer support is more effective personal recovery when delivered as an adjunct to existing mental health services (Høgh Egmose et al. 2023). Within emerging PAT models, PSWs may be positioned in complementary or co‐facilitated roles alongside clinical teams. Nevertheless, empirical work examining PSW roles within PAT specifically remains limited.

The qualitative results (see Supplementary Material 1) showed that PSWs feel “under‐utilized and under‐involved” yet willing to provide ongoing support, facilitate integration, and ensure continuity of care. Future implementation models need to carefully consider the role of multidisciplinary therapists and supporters—including the role of PSWs—and their potential to be involved in more comprehensive care that enhances post‐integration support within a safe, ethical clinical governance framework. Traditional Indigenous practices similarly emphasize community‐based guidance beyond the acute experience (Dorsen et al. 2025; George et al. 2022). These parallels underscore the enduring value of sustained, community‐grounded support in psychedelic interventions.

This principle of community‐based integration now influences policy in the United States. Oregon's regulated psilocybin services have created pathways for non‐licensed peer specialists, often tasked with case management or co‐facilitating groups (Skiles et al. 2023). Such precedents suggest similar roles may be considered when integrating Australia's established PSW workforce into PAT frameworks. Consistent with this, our findings highlight the importance of workforce development and role clarity when considering the future integration of lived‐experience‐informed roles within regulated PAT models. As frameworks develop, ensuring equitable access and safe integration of non‐clinical roles will be essential.

## 5 Conclusion

In conclusion, this study offers the first insight into PSW attitudes toward PAT in Australia, identifying a non‐clinician workforce that is broadly supportive and optimistic about its potential. Optimism was linked to lived experience and greater perceived knowledge, yet tempered by concerns around treatment costs, training accessibility, and role clarity. As Australia moves toward wider implementation, developing tailored education pathways and structured models for integration will be critical to ensuring PSWs are equipped to contribute effectively and responsibly to emerging PAT frameworks.

## Author Contributions

**Aloysius Amos Lau**: writing – review and editing, writing – original draft, conceptualization, methodology, formal analysis, investigation, data curation, project administration. **Boen Raner‐galutera**: investigation, writing – original draft, data curation, methodology, formal analysis, writing – review and editing. **Siegfried Lichtwark**: conceptualization, investigation, writing – review and editing, methodology, validation, project administration, supervision.

## Funding

The authors have nothing to report.

## Ethics Statement

Ethics approval for this study was obtained from the Eastern Health Human Research Ethics Committee (Reference No: E24‐001‐105296). All participants provided informed consent prior to completing the online survey and were advised that participation was voluntary and responses were anonymized.

## Conflicts of Interest

The authors declare no conflicts of interest related to this work. The study was conducted independently and received no specific funding from any commercial or financial organization that could be construed as a potential conflict of interest.

## Supporting information




**Supplementary Materials**: brb371521‐sup‐0001‐SuppMat.docx

## Data Availability

The participants of this study did not give written consent for their data to be shared publicly, so due to the sensitive nature of the research, supporting data is not available.
