# Supplementary material for: A Missing Voice? Peer Support Workers’ Perceptions of Psychedelic‐Assisted Therapy in Australia: A Cross‐Sectional Survey
Source: Brain Behav. 2026 May 31;16(6):e71521. doi: 10.1002/brb3.71521 (PMC13239322; doi:10.1002/brb3.71521)
Supplement: Supplementary file 1 — Supplementary Materials: brb371521‐sup‐0001‐SuppMat.docx [file BRB3-16-e71521-s001.docx]

**Supplementary Table 1**: Distribution of Responses to Survey Items

| **Survey Item** | **Strongly Disagree** | **Disagree** | **Neutral** | **Agree** | **Strongly Agree** |
| --- | --- | --- | --- | --- | --- |
| I am cautious of endorsing PAT as there is not enough research evidence. | 4  (12.1%) | 9  (27.3%) | 10  (30.3%) | 6  (18.2%) | 4  (12.1%) |
| I believe PAT will increase the risk for subsequent psychiatric disorders. | 4  (12.1%) | 16  (48.5%) | 9  (27.3%) | 4  (12.1%) | 0  (0.0%) |
| I believe PAT increases the risk of long-term cognitive impairment. | 9  (27.3%) | 12  (36.4%) | 10  (30.3%) | 2  (6.1%) | 0  (0.0%) |
| I believe PAT reduces the risk for the development of subsequent substance use disorders. | 1  (3.0%) | 6  (18.2%) | 12  (36.4%) | 12  (36.4%) | 2  (6.1%) |
| I am concerned that I would not be able to relate to the experiences of patients who have undergone PAT. | 12  (36.4%) | 11  (33.3%) | 4  (12.1%) | 4  (12.1%) | 2  (6.1%) |
| I believe the use of psychedelics in recreational settings is unsafe. | 8  (24.2%) | 6  (18.2%) | 7  (21.2%) | 8  (24.2%) | 4  (12.1%) |
| I believe that even under medical supervision, the use of psychedelics is unsafe. | 13  (39.4%) | 10  (30.3%) | 8  (24.2%) | 2  (6.1%) | 0  (0.0%) |
| There is a lack of trained PAT providers. | 0  (0.0%) | 0  (0.0%) | 7  (21.2%) | 10 (30.3%) | 15  (45.5%) |
| I believe the current training available for PAT is inadequate. | 1  (3.0%) | 1  (3.0%) | 16  (48.5%) | 5  (15.2%) | 9  (27.3%) |
| I am concerned about the potential risks and burdens of PAT. | 1  (3.0%) | 11  (33.3%) | 4  (12.1%) | 13 (39.4%) | 3  (9.1%) |
| I have encountered great interest from the patients I support about PAT. | 2  (6.1%) | 2  (6.1%) | 14  (42.4%) | 10 (30.3%) | 4  (12.1%) |
| Recommending PAT goes against my spiritual beliefs. | 19  (57.6%) | 7  (21.2%) | 4  (12.1%) | 2  (6.1%) | 0  (0.0%) |
| I/my patients are concerned about the cost/insurance coverage for PAT. | 0  (0.0%) | 1  (3.0%) | 14  (42.4%) | 8  (24.2%) | 9  (27.3%) |
| I believe PAT will be inaccessible to those most in need. | 0  (0.0%) | 1  (3.0%) | 9  (27.3%) | 13 (39.4%) | 9  (27.3%) |
| Greater knowledge about PAT will increase my willingness to recommend it to the patients I support. | 0  (0.0%) | 0  (0.0%) | 4  (12.1%) | 11 (33.3%) | 17  (51.5%) |
| I believe with the current experience I have, I am capable of supporting patients who have undergone PAT. | 2  (6.1%) | 9  (27.3%) | 5  (15.2%) | 9  (27.3%) | 8  (24.2%) |
| I am confident that with the right training and guidelines, I am capable of supporting patients who have undergone PAT. | 2  (6.1%) | 2  (6.1%) | 1  (3.0%) | 10 (30.3%) | 18  (54.5%) |
| I am confident that PAT will be effective in treating mental health disorders. | 0  (0.0%) | 0  (0.0%) | 7  (21.2%) | 13 (39.4%) | 13  (39.4%) |
| It is very likely that PAT will become a standard practice in our field. | 0  (0.0%) | 3  (9.1%) | 9  (27.3%) | 10 (30.3%) | 11  (33.3%) |
| I am confident in recommending PAT to the patients I am supporting. | 5  (15.2%) | 7  (21.2%) | 9  (27.3%) | 9  (27.3%) | 3  (9.1%) |
| I believe PAT will be more/just as effective as existing treatments in mental health disorders. | 1  (3.0%) | 0  (0.0%) | 10  (30.3%) | 11 (33.3%) | 11  (33.3%) |
| If I had the opportunity, I would like to receive training to support patients undergoing PAT. | 2  (6.1%) | 0  (0.0%) | 0  (0.0%) | 6  (18.2%) | 25  (75.8%) |
| Compared to my peers and other professionals in my sector, I consider myself more optimistic about the potential of PAT. | 1  (3.0%) | 3  (9.1%) | 5  (15.2%) | 12  (36.4%) | 12  (36.4%) |
| I have a strong understanding of the differences among each of the psychedelic substances. | 2  (6.1%) | 6  (18.2%) | 8  (24.2%) | 8  (24.2%) | 9  (27.3%) |
| I have a strong understanding of the different effects among each of the psychedelic substances. | 2  (6.1%) | 5  (15.2%) | 7  (21.2%) | 13  (39.4%) | 6  (18.2%) |
| I am very knowledgeable about scientific literature around PAT. | 6  (18.2%) | 10  (30.3%) | 6  (18.2%) | 10  (30.3%) | 1  (3.0%) |
| I have a strong understanding of how PAT works. | 5  (15.2%) | 6  (18.2%) | 12  (36.4%) | 8  (24.2%) | 2  (6.1%) |
| I have a strong understanding of who should be excluded from PAT. | 6  (18.2%) | 11  (33.3%) | 5  (15.2%) | 10  (30.3%) | 1  (3.0%) |
| There are no disadvantages in PAT. | 6  (18.2%) | 15  (45.5%) | 9  (27.3%) | 2  (6.1%) | 1  (3.0%) |
| PAT deserves further research as a potential treatment in mental health. | 1  (3.0%) | 0  (0.0%) | 2  (6.1%) | 4  (12.1%) | 26  (78.8%) |
| I have a strong interest in PAT. | 1  (3.0%) | 0  (0.0%) | 1  (3.0%) | 9  (27.3%) | 22  (66.7%) |
| I have an interest in supporting patients who have had PAT. | 0  (0.0%) | 0  (0.0%) | 2  (6.1%) | 10  (30.3%) | 21  (63.6%) |
| Psychedelics should be illegal for recreational use. | 14  (42.4%) | 9  (27.3%) | 6  (18.2%) | 3  (9.1%) | 1  (3.0%) |
| Peer support workers should have a personal experience with psychedelics before supporting patients who have undergone PAT. | 5  (15.2%) | 7  (21.2%) | 2  (6.1%) | 12  (36.4%) | 7  (21.2%) |
| Endorsing PAT will have negative impacts on my reputation at work. | 9  (27.3%) | 9  (27.3%) | 9  (27.3%) | 6  (18.2%) | 0  (0.0%) |
| Endorsing PAT will have negative impacts on my reputation in my personal life. | 13  (39.4%) | 6  (18.2%) | 9  (27.3%) | 4  (12.1%) | 1  (3.0%) |
| The endorsement of PAT goes against the ideologies of those around me at work. | 6  (18.2%) | 13  (39.4%) | 9  (27.3%) | 5  (15.2%) | 0  (0.0%) |
| The endorsement of PAT goes against the ideologies of those around me in my personal life. | 8  (24.2%) | 9  (27.3%) | 9  (27.3%) | 7  (21.2%) | 0  (0.0%) |
| My colleagues at work would support the implementation of PAT. | 1  (3.0%) | 4  (12.1%) | 13  (39.4%) | 11 (33.3%) | 4  (12.1%) |
| Management at work would support the implementation of PAT. | 4  (12.1%) | 8  (24.2%) | 11  (33.3%) | 7  (21.2%) | 3  (9.1%) |
| In my social circle, friends/family would support the implementation of PAT. | 1  (3.0%) | 3  (9.1%) | 8  (24.2%) | 13 (39.4%) | 8  (24.2%) |
|  | | | | | |
| **Desired Educational Topics** | **Strongly Disagree** | **Disagree** | **Neutral** | **Agree** | **Strongly Agree** |
| Pharmacology of psychedelics. | 0  (0.0%) | 1  (3.3%) | 2  (6.7%) | 16  (53.3%) | 11  (36.6%) |
| Side effects of psychedelics. | 0  (0.0%) | 0  (0.0%) | 4  (13.3%) | 10  (33.3%) | 16  (53.3%) |
| Management of "bad trips”. | 0  (0.0%) | 0  (0.0%) | 2  (6.7%) | 8  (26.7%) | 20  (66.7%) |
| Contraindications for PAT. | 0  (0.0%) | 0  (0.0%) | 4  (13.3%) | 10  (33.3%) | 16  (53.3%) |
| Potential benefits of PAT. | 0  (0.0%) | 0  (0.0%) | 4  (13.3%) | 12  (40%) | 14  (46.7%) |

**Supplementary Table 2**: Distribution of Responses to Survey Items relating to Personal Psychedelic Experience (n = 23)

| **Survey Item** | **Strongly Disagree** | **Disagree** | **Neutral** | **Agree** | **Strongly Agree** |
| --- | --- | --- | --- | --- | --- |
| I ensured that the environment around me was ideal for a positive experience. | 1  (4.3%) | 4  (17.4%) | 4  (17.4%) | 6  (26.1%) | 8  (34.8%) |
| I ensured that my internal state of mind was ideal for a positive experience. | 2  (8.7%) | 6  (26.1%) | 3  (13.0%) | 5  (21.7%) | 7  (30.4%) |
| The experience was spiritually significant. | 0  (0.0%) | 3  (13.0%) | 5  (21.7%) | 8  (34.8%) | 7  (30.4%) |
| The experience was personally meaningful. | 1  (4.3%) | 0  (0.0%) | 2  (8.7%) | 11  (47.8%) | 9  (39.1%) |
| The experience has had long-lasting positive effects on my life. | 0  (0.0%) | 4  (17.4%) | 2  (8.7%) | 6  (26.1%) | 11  (47.8%) |

**Supplementary Figure 1**: The topics found in the literature review and the structure of the relevant questions used in the interview.


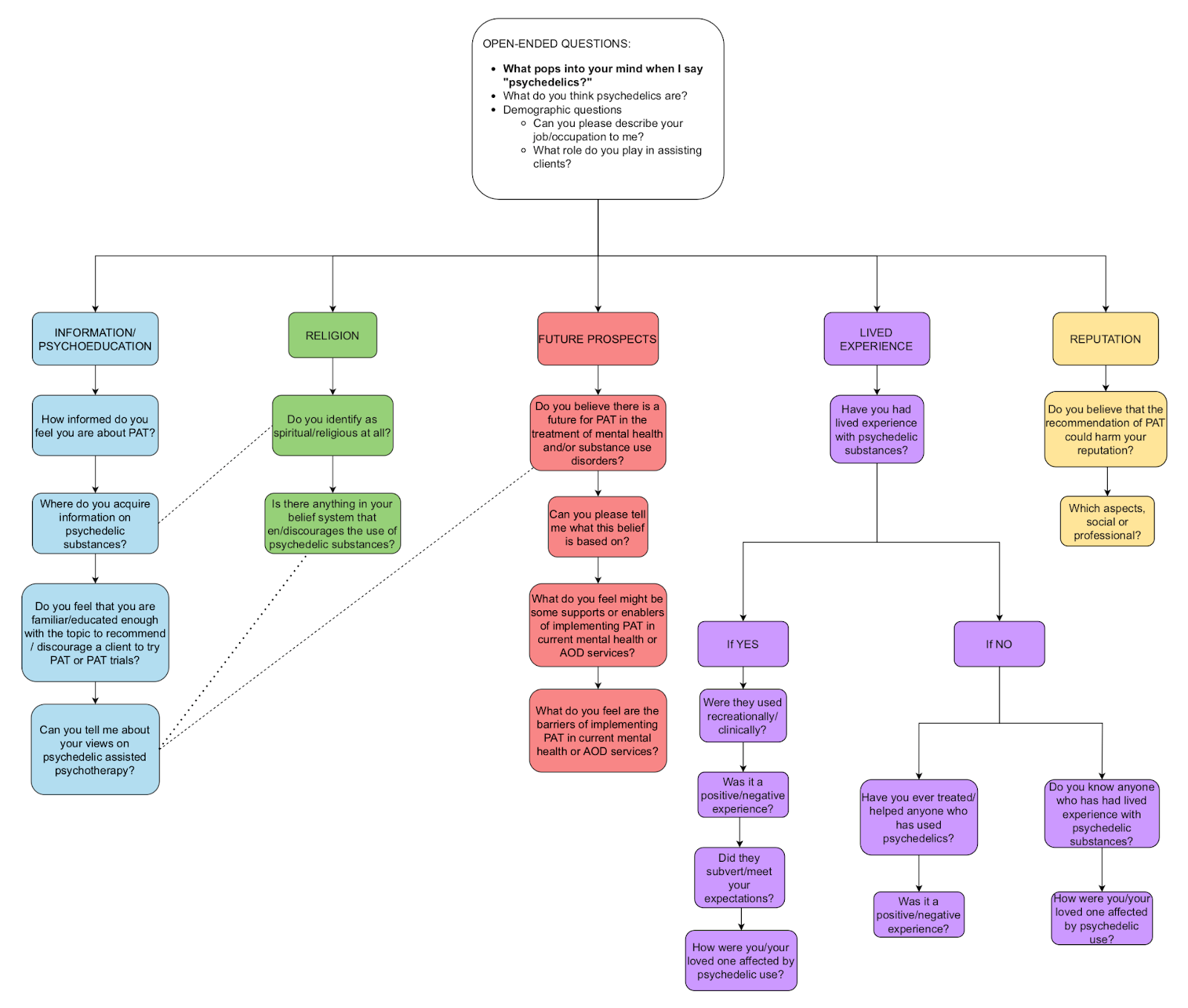


**Supplementary Material 1**: Qualitative Arm of the study

A review of the qualitative literature revealed five key topics that influence how various health professionals (such as palliative care workers, clinicians and psychiatrists) perceive PAT. These topics included information/psychoeducation, spirituality/religion, future prospects, client safety, lived experience and reputation. These topics formed the basis for the line of questioning during the semi-structured interview, which included open- and close-ended questions exploring each topic (**See Supplementary Figure 1**). Participants were survey respondents of our study who opted into their interview participation. Interviews were conducted via videoconference, audio recorded and transcribed verbatim using the in-built Zoom software. The transcribed responses were reviewed by the interviewer (BG, student researcher) for fidelity to the recorded interview and discrepancies were corrected to ensure the accurate representation of the participants’ words. The interviews ranged from approximately 30 minutes to one hour in duration.

After the transcription of the interviews was complete, each participant’s response was de-identified. The responses were analysed using a directed approach to qualitative content analysis, building upon the framework of the quantitative arm. A summative approach to qualitative analysis was also used to categorise and analyse the various themes and patterns (Hsieh & Shannon, 2005). The research team rigorously discussed the different themes each member encountered to ensure consistency throughout the qualitative analysis.

Of the five interviewees, three identified as male and two as female.

The directed content analysis of the interview transcripts resulted in categorisation of the content into five topics, three of which were previously identified in the literature review. These topics, in order of most mentioned were lived experience (which appeared in 5/5 interviews), stigma (5/5), accessibility (4/5), knowledge/psychoeducation (3/5) and client safety/sovereignty (3/5). Of these lived experience, stigma and knowledge/psychoeducation are topics that influence perceptions towards PAT previously identified in the literature [(Barnett et.al, 2021; Sholevar et al., 2023)](https://www.zotero.org/google-docs/?vNDXhi), two new topics were found: accessibility of PAT and client safety/sovereignty. Participants identified most of these topics as mitigating factors that may hinder the implementation of PAT, with two individuals noting that, since each client is unique, they would merely function as a “sounding bell” (Participant A) and would only discuss PAT with a client if the client broached the topic first (client safety/sovereignty). One individual felt as though recommending PAT was a “moot point” as “anybody that [they might] suggest it to could not afford it” (accessibility topic) (Participant D).

The interview participants’ concerns around client safety and treatment accessibility were corroborated by strong agreement in survey responses to items on accessibility, with 69.7% of participants indicating that cost or (lack of) insurance coverage would be a barrier to PAT, and 66.7% agreeing that PAT would be inaccessible to those most in need.

Yet, despite stigma, safety concerns, accessibility issues and low levels of psychoeducation in clients, all PSWs that were interviewed held strong interest in PAT. They expressed optimism for the future of PAT, noting that the method of therapy will “100%…take off!” (Participant C). Interviewees discussed the greater need for PSWs to be involved in the delivery of PAT. There was a consensus that the expertise of individuals with a “psychonaut[ic]” understanding of the experience; where the peaks may occur and how the cooldown should be facilitated, would be beneficial for the implementation of the therapy (Participants C and E). Participants felt that the lived experience workforce was being severely “under-utilised and under-involved” and should be involved in policy, clinical trials and the administration of PAT for the client to have the best care possible (Participant E).

Accessible education pathways for non-clinician roles emerged as a critical topic. While previous studies have established therapist competencies as essential for effective PAT delivery [(Phelps, 2017)](https://www.zotero.org/google-docs/?c7YBdk), current training programs remain geared towards licensed clinical health professionals, excluding non-clinician roles. Integrating PAT-specific content into PSW educational programs could enhance the preparedness and impact of this workforce in supporting PAT interventions.

Limitations of the qualitative arm of this study included the relatively small number of interviewees (n=5) as this has the potential to restricts data variability and thus limits capture of diverse PSW perspectives. The self-selecting nature of the recruitment for the interview could lead to a positive screw of the information received as all PSWs who responded were already somewhat biased towards PAT as the lack of critical voices demonstrates. Collectively, these factors highlight the exploratory nature of our findings and the need for future, larger-scale investigations into non-clinician roles in PAT delivery.

**Supplementary Material 2**: Peer Support Worker Survey Questionnaire

**Section 1: Demographic Measures**

1. Are you currently employed as a Peer Support Worker? (Yes/No)
2. What is your age?
3. How would you describe your gender:

- Male or Man
- Female or Woman
- Non-binary / third gender
- I use a different term (Please Specify)
- Prefer not to say

1. How would you describe your sexuality:

- Straight or heterosexual
- Lesbian, gay, or homosexual
- Bisexual
- Queer
- Other (Please Specify)
- Prefer not to say

1. Do you identify as Aboriginal or Torres Strait Islander? (Yes/No)
2. What is your highest level of education completed?
   - Primary school
   - Secondary school
   - Trade/ apprenticeship
   - TAFE/ diploma
   - Undergraduate degree
   - Masters degree
   - Doctorate/ PhD
3. Please enter the postcode of your place of work. This information will help us understand the geographical distribution of our respondents.
4. Please indicate the level of experience you have as a Peer Support Worker.
   - Less than 6 months
   - Between 6-12 months
   - Between 1-3 years
   - More than 3 years
5. What is your primary daily practice activity?
   - Client Engagement
   - Education
   - Research
   - Management
   - Public Health Advocacy
6. Do you identify as having lived and/or living experience? (Yes/No)

*(If yes to Q10, participant to answer Q11 and Q12)*

1. Do you have lived and/or living experience with Mental Health problems? (Yes/No)
2. Do you have lived and/or living experience with Alcohol or Other Drug problems? (Yes/No)
3. Do you identify as a religious/spiritual individual? (Yes/No)

*(If yes to Q13, participant to answer Q14)*

1. Please select the option that best describes your beliefs.
   - Christianity
   - Islam
   - Hinduism
   - Buddhism
   - Sikhism
   - Judaism
   - Other Religion (Please Specify)
   - Spiritual but not affiliated with a specific religion
   - Agnostic
   - Prefer not to say

**Information**

The following questions seek to gather information about your knowledge and attitudes towards the use of psychedelic substances and their use in the treatment of mental health and substance use disorders.

Psychedelics, also known as hallucinogens, are psychoactive substances that can alter one’s perception, mood and thought patterns. Examples of such substances include psilocybin (magic mushrooms), LSD (acid),

ayahuasca, mescaline, DMT and 2C-B. Other non-classical psychedelics including ketamine and MDMA (molly) will be included in this definition for the purposes of this survey. The questions may highlight your opinions towards psychedelics or psychedelic assisted therapy (PAT). We understand that you may or may not have personal experiences with psychedelics or be fully aware of the opinions held by your organisation, colleagues, or clients regarding this matter. We would like to reassure you that your responses will be kept confidential and your personal details de-identified. We ask that you respond truthfully and to the best of your ability.

**Section 2: Knowledge**

1. What is your main source(s) of information on psychedelic-assisted therapy?

- Journal articles
- News reports
- Private organisations
- Mainstream media
- Social media
- Colleagues/Peers
- Other (Please Specify)

Please indicate your level of agreement to the following statements about Psychedelic Assisted Therapy (PAT):

|  | Strongly Disagree | Disagree | Neutral | Agree | Strongly Agree |
| --- | --- | --- | --- | --- | --- |
| 16. I have a strong understanding of the differences among each of the psychedelic substances. | o | o | o | o | o |
| 17. I have a strong understanding of the different effects of psychedelic effects among each of the psychedelic substances. | o | o | o | o | o |
| 18. I am very knowledgeable about scientific literature around PAT. | o | o | o | o | o |
| 19. I have a strong understanding of how PAT works. | o | o | o | o | o |
| 20. I have a strong understanding of who should be excluded from PAT. | o | o | o | o | o |
| 21. There are no disadvantages in PAT. | o | o | o | o | o |
| 22. PAT deserves further research as a potential treatment in mental health. | o | o | o | o | o |

**Section 3: Social/professional role and identity**

|  | Strongly Disagree | Disagree | Neutral | Agree | Strongly Agree |
| --- | --- | --- | --- | --- | --- |
| 23. Endorsing PAT will have negative impacts on my reputation at work. | o | o | o | o | o |
| 24. Endorsing PAT will have negative impacts on my reputation in my personal life. | o | o | o | o | o |
| 25. The endorsement of PAT goes against the ideologies of those around me at work. | o | o | o | o | o |
| 26. The endorsement of PAT goes against the ideologies of those around me in my personal life. | o | o | o | o | o |
| 27. My colleagues at work would support the implementation of PAT. | o | o | o | o | o |
| 28. Management at work would support the implementation of PAT. | o | o | o | o | o |
| 29. In my social circle, friends/family would support the implementation of PAT. | o | o | o | o | o |

**Section 4: Belief and Optimism**

|  | Strongly Disagree | Disagree | Neutral | Agree | Strongly Agree |
| --- | --- | --- | --- | --- | --- |
| 30. I believe with the current experience I have, I am capable of supporting patients who have undergone PAT. | o | o | o | o | o |
| 31. I am confident that with the right training and guidelines, I am capable of supporting patients who have undergone PAT. | o | o | o | o | o |
| 32. I am confident that PAT will be effective in treating mental health disorders. | o | o | o | o | o |
| 33. It is very likely that PAT will become a standard practice in our field. | o | o | o | o | o |
| 34. I am confident in recommending PAT to the patients I am supporting. | o | o | o | o | o |
| 35. I believe PATwill be more/just as effective as existing treatments in mental health disorders. | o | o | o | o | o |
| 36. If I had the opportunity, I would like to receive training to support patients undergoing PAT. | o | o | o | o | o |
| 37. Compared to my peers and other professionals in my sector, I consider myself more optimistic about the potential of PAT. | o | o | o | o | o |

**Section 5: Beliefs about Consequences**

|  | Strongly Disagree | Disagree | Neutral | Agree | Strongly Agree |
| --- | --- | --- | --- | --- | --- |
| 38. I am cautious of endorsing PAT as there is not enough research evidence. | o | o | o | o | o |
| 39. I believe PAT will increase the risk for subsequent psychiatric disorders. | o | o | o | o | o |
| 40. I believe PAT increases the risk of long-term cognitive impairment. | o | o | o | o | o |
| 41. I believe PAT reduces the risk for the development of subsequent substance use disorders. | o | o | o | o | o |
| 42. I am concerned that I would not be able to relate to the experiences of patients who have undergone PAT. | o | o | o | o | o |
| 43. I believe the use of psychedelics in recreational settings is unsafe. | o | o | o | o | o |
| 44. I believe that even under medical supervision, the use of psychedelics is unsafe. | o | o | o | o | o |

**Section 6: Motivations and Goals**

|  | Strongly Disagree | Disagree | Neutral | Agree | Strongly Agree |
| --- | --- | --- | --- | --- | --- |
| 45. I have a strong interest in PAT. | o | o | o | o | o |
| 46. I have an interest in supporting patients who have had PAT. | o | o | o | o | o |
| 47. Psychedelics should be illegal for recreational use. | o | o | o | o | o |
| 48. Peer support workers should have a personal experience with psychedelics before supporting patients who have undergone PAT. | o | o | o | o | o |

**Section 7: Personal Experience with Psychedelics**

The following questions aim to understand your experiences and the experiences of those around you with regards to the use of psychedelics. This could be a personal experience from taking psychedelics, watching or hearing about an experience from a friend or family member. We would like you to comment on the most significant experience you have had in each of the following questions.

1. Have you cared for patients who have had an experience using psychedelics? (Yes/No)

*(If yes to Q49, participant to answer Q50)*

1. From your understanding of their experience, how would you rate its effect on their sense of well-being and life satisfaction?

| Extremely Negative | Neutral | Extremely Positive |
| --- | --- | --- |

| o | o | o | o | o | o | o | o | o | o | o |
| --- | --- | --- | --- | --- | --- | --- | --- | --- | --- | --- |

1. Do you have family and/or friends who have had an experience using psychedelics? (Yes/No)

*(If yes to Q51, participant to answer Q52)*

1. From your understanding of their experience, how would you rate its effect on their sense of well-being and life satisfaction?

| Extremely Negative | Neutral | Extremely Positive |
| --- | --- | --- |

| o | o | o | o | o | o | o | o | o | o | o |
| --- | --- | --- | --- | --- | --- | --- | --- | --- | --- | --- |

1. Have you ever had a personal experience using psychedelics? (Yes/No)

*(If yes to Q53, participant to answer Q54-Q59)*

1. From this experience, how would you rate its effect on your sense of well-being and life satisfaction?

| Extremely Negative | Neutral | Extremely Positive |
| --- | --- | --- |

| o | o | o | o | o | o | o | o | o | o | o |
| --- | --- | --- | --- | --- | --- | --- | --- | --- | --- | --- |

With consideration of your experience as a whole, please indicate your level of agreement to the following statements:

|  | Strongly Disagree | Disagree | Neutral | Agree | Strongly Agree |
| --- | --- | --- | --- | --- | --- |
| 55. I ensured that the environment around me was ideal for a positive experience. | o | o | o | o | o |
| 56. I ensured that my internal state of mind was ideal for a positive experience. | o | o | o | o | o |
| 57. The experience was spiritually significant. | o | o | o | o | o |
| 58. The experience was personally meaningful. | o | o | o | o | o |
| 59. The experience has had long lasting positive effects on my life. | o | o | o | o | o |

**Section 8: Barriers and Facilitators**

|  | Strongly Disagree | Disagree | Neutral | Agree | Strongly Agree |
| --- | --- | --- | --- | --- | --- |
| 60. There is a lack of trained PAT providers. | o | o | o | o | o |
| 61. I believe the current training available for PAT is inadequate. | o | o | o | o | o |
| 62. I am concerned about the potential risks and burdens of PAT. | o | o | o | o | o |
| 63. I have encountered great interest from the patients I support about PAT. | o | o | o | o | o |
| 64. Recommending PAT goes against my spiritual beliefs. | o | o | o | o | o |
| 65. I/my patients are concerned about the cost/insurance coverage for PAT. | o | o | o | o | o |
| 66. I believe PAT will be inaccessible to those most in need. | o | o | o | o | o |
| 67. Greater knowledge about PAT will increase my willingness to recommend it to the patients I support. | o | o | o | o | o |

**Section 9: Desired Educational Topics**

|  | Strongly Disagree | Disagree | Neutral | Agree | Strongly Agree |
| --- | --- | --- | --- | --- | --- |
| 68. Pharmacology of psychedelics | o | o | o | o | o |
| 69. Side effects of psychedelics | o | o | o | o | o |
| 70. Management of “bad trips” | o | o | o | o | o |
| 71. Contraindications for PAT | o | o | o | o | o |
| 72. Potential benefits of PAT | o | o | o | o | o |

**Section 10: Qualitative Responses**

The next few questions give you an opportunity to write open-ended responses to things we may not have explored adequately during the survey. They aim to gather qualitative insights through open-ended questions to give us a comprehensive view of the factors that may influence your attitudes toward research recruitment for psychedelic assisted therapy trials.

1. Please write down the main factors that would make you hesitant in recommending someone for research recruitment.
2. What resources or changes would most improve your confidence in recommending research study recruitment to potential participants?
